# Associated factors of REM sleep without atonia in younger (≤ 50 years) hospitalized psychiatric patients

**DOI:** 10.1186/s12888-020-02879-4

**Published:** 2020-10-01

**Authors:** Jitka Bušková, Eva Miletínová, Monika Kliková, Martin Bareš, Tomáš Novák, Jiřina Kosová, Pavla Stopková, Jana Kopřivová

**Affiliations:** 1 Department of Sleep Medicine, National Institute of Mental Health, Topolová 748, 250 67 Klecany, Czech Republic; 2 Third Faculty of Medicine, Charles University, Prague, Czech Republic

**Keywords:** REM sleep without atonia, REM sleep behaviour disorder, Psychiatric patients, Antidepressants, Antipsychotics, Anxiety, Depression

## Abstract

**Background:**

Isolated REM sleep without atonia (RSWA) as a main polysomnograhic feature of REM sleep behaviour disorder (RBD) is thought to be a prodromal or subclinical state of the disease. RSWA/RBD occurence in psychiatric population is much more frequent than in general population but its associated factors are still not known.

**Methods:**

We invited 88 psychiatry in-patients to undervent video-polysomnography. The visual scoring was focused on RSWA in submentales and flexores digitales superficiales muscles. This parametr was subsequently correlated mainly with age/gender, their medication and mental status.

**Results:**

The RWSA was mostly still in normal range despite the fact, that selected psychiatry patients (≤ 50 years) were taking several classes of psychoactive medication. 3,6% had convincingly RBD, although 35.7% reported rare lifetime occurence of dream-enacting behaviour and 62.8% sporadic nightmares. We found correlation between RSWA and SNRI medication class (*p* = 0.015), specifically venlafaxine (*p* = 0.029) as well as quetiapine (*p* = 0.030). Another significant associated factors were current anxiety (*p* < 0.001) and depressive symptoms (*p* = 0.05), but we found no relation between RSWA and given diagnosis.

**Conlucions:**

Isolated RSWA in younger psychiatry patients might be a result of multiple factors, including medication and current mental status but these factors are in most cases not sufficient to manifest RBD.

## Background

REM sleep without atonia (RSWA) is defined as excessive sustained or intermittent elevation of chin electromyographic (EMG) tone or excessive phasic chin or limb EMG twitching [1]. Together with dream-enacting behaviour (DEB) RSWA is a key polysomnograhic feature of REM sleep behaviour disorder (RBD). Two major clinical aspects are important in RBD. Firstly, the nocturnal behaviour may result in severe injuries for the patient or his/her bed partner. Secondly, the idiopathic form of RBD represents an early-stage of α-synucleinopathy [2, 3], which emphasizes timely recognition of the disease, especially in terms of future neuroprotective therapy. Isolated RSWA is hypothesized to be a prodromal or subclinical phase of RBD, but its relevance is still unknown, especially in psychiatric population.

There is some evidence that RBD in psychiatric patients is much more frequent than in the general population [4, 5]. Likewise, there are several studies showing close association of RBD/RSWA with psychiatric illness or with antidepressant use [5–8]. However, there is still ongoing controversy over whether RBD in psychiatric patients is one of the separate subgroups of RBD patients, and if so, if it is solely related to the effects of mental illness per se or antidepressants, or to the combination of both factors /other clinical factors [6, 7, 9, 10].

The main aims of our study were 1) to find out if isolated RSWA depends on age and gender in psychiatric patients; or wheather another clinical factors might play a role, 2) to delineate the influence of medication, given diagnosis and current mental stutus on the degree of RSWA. According to the presumption that RSWA could be induced by the use of antidepressants, especially SSRI/SNRI [6, 8, 11–14], we hypothesized that the RSWA would be more prominent on this medication classes compared to other medical classes and will be more distinct in patients with severe psychiatric symptoms, independently of age and gender.

## Methods

### Subjects

Psychiatric in-patients hospitalized for 6–8 weeks between June 2018 and December 2019 at the National Institute of Mental Health, CZ, were invited to participate in this prospective polysomnography study. At the day of video-polysomnography (v-PSG), all of them completed the BDI-II [15] and BAI [16] questionnnaires. They underwent clinical examinations by a neurologist and a psychiatrist in order to exclude any disease that could be potentially associated with RBD, such as narcolepsy, dementia and Parkinson’s disease. Along with sociodemographic data (age, gender, education), we documented sleep comorbidities (that were consequently confirmed by video-polysomnography). All the patients were specifically asked about dream-enacting behaviour and nightmares. From other clinical factors, history taking at the day of v-PSG, was specifically focused on their family history of mental illness and perinatal history, autoimmune disease and smoking/alcohol/drugs use. Given psychiatric diagnosis and the use of psychoactive medications was listed.

### Video-polysomnography

Nocturnal video-polysomnography (v-PSG) was performed using a digital Brainscope PSG system and consisted of 19-channel electroencephalography (EEG), electrooculography (EOG), electromyography of the bilateral mentalis muscle, the bilateral flexor digitorum superficialis muscle (FDS) and the bilateral tibialis anterior muscle (TA), electrocardiography, nasal pressure, nasal and oral air flow, thoracic and abdominal respiratory effort, oxygen saturation, microphone and synchronized video monitoring from 10 p.m. to 6 a.m. according to the recommendation by the American Academy of Sleep Medicine (AASM) [1]. All of the features were analysed visually. Sleep stages, arousals, respiratory events, and limb movements were scored according to the AASM Manual for the Scoring of Sleep and Associated Events, version 2.22015 (Scoring Manual 2007) [1, 17] with an exception for the REM sleep rules allowing to score REM sleep stage despite the prominent EMG activity in the mentalis muscle channel [18]. The occurence of the first rapid eye movements (REMs) in the EOG channel was used to determine the onset of particular REM sleep period. The end of each REM sleep period was determined when either no REMs were detected in 3 consecutive minutes or awakening or K complexes or sleep spindles were observed.

We only included subjects with > 5 min of REM sleep. RSWA was scored in the submentalis muscle and bilateral FDS channels in 1-s mini-epochs. All artefacts and increases in EMG tone due to arousals from respiratory events, periodic limb movements (PLM) events and any kind of arousal-related movement were excluded as much as possible from the quantitative scoring before the analysis of EMG activity. RSWA was visually scored in 30s epochs: either increased tonic activity, defined as an increase of > 50% in baseline EMG amplitude (measured during nonREM sleep) or > 10 μV during > 50% of REM epoch, or increased phasic activity, defined as all muscle activity during 0,1–5 s that exceeded 4 times the background EMG activity in 1 s-mini-epochs within a 30s REM epoch. Each 1-s mini-epoch was scored as having or not having “any” EMG activity, irrespective of containing phasic or tonic EMG activity. After scoring any EMG activity in the submentalis muscle channels, we scored the EMG activity in the FDS channels in 1-s mini-epochs not having any EMG activity already scored in the submentalis muscle channels. Thus, we obtained an overall score expressing the percentage of disturbed muscle tone in REM sleep referring to the total amount of REM sleep-related EMG activity (RSWA %). We did not quantify REM sleep behavioural events and we did not established specific cut-off for RBD.

The study was approved by the local Ethics Committee. All of the patients signed the informed consent before inclusion in the study.

### Statistical analysis

Descriptive parameters, such as demographic data, frequency of particular diagnosis and types of medication were calculated for particular variables of interest. After testing for normality by using *Shapiro-Wilk test,* we used non-parametric correlations and *Kruskal-Wallis test* for comparing multiple groups*.* All of the tests were conducted by using *SPSS-23* program.

## Results

From 120 invited psychiatry in-patients, total of 88 agreed to participate in the study. We excluded 8 patients because of the records’ artefacts (biological-muscle, respiratory, ECG or technical), 12 records for obstructive sleep apnoea (AHI ≥ 15) and 10 periodic limb movements in sleep (PLMI ≥15) mimicking RBD or not allowing for the evaluation of muscle tone in REM sleep, and 2 records with REM sleep under 5 min. Finaly, 56 patients (42 F/14 M, mean age 38.25, age range 26–50 years) were included in our study. The Table 1 describes polysomnographic findings in all patients. In these recordings we evaluated REM sleep muscle tone. The mean isolated RSWA was 8.77%, SD = 9.23. This was a baseline for all the planned analysis. Two patients (3.6%) actually passed the criteria for RBD (with prominent dream-enacting behavior on video-polysomnography and with the percentage of disturbed muscle tone in REM sleep to be 32.5 and 55%). In the other patients we did not capture DEB immediately on video-polysomnography, but 35.7% reported lifetime occurence of DEB (rare/once or twice a life) and 68.2% refered nightmares. We did not find any correlation between age/gender and total RSWA%.

**Table 1:** Polysomnography parameters of psychiatry patients

Variable	Psychiatric patients (*n* = 56)
**TST (min)**	359 (SD = 85.78)
**SOL (min)**	30.60 (SD = 24.86)
**SE %**	85.62 (SD = 6.78)
**WASO %**	14.36 (SD = 10.37)
**NREM 1%**	5.5 (SD = 5.60)
**NREM 2%**	47.79 (SD = 10.15)
**NREM 3%**	17.57 (SD = 8.22)
**AHI**	9.16 (SD = 9.09)
**PLMI**	12.45 (SD = 11.03)
**REM %**	14.78 (SD = 6.67)
**REM latency**	172.35 (SD = 72.58)
**REM periods**	2–5
**RSWA %**	8.77 (SD = 9.23)

Legend: *TST* total sleep time, *SOL* sleep onset latency, *SE* sleep efficiency, *WASO* wake after sleep onset, *NREM* non-REM sleep, *AHI* apnoea-hypopnoea index, *PLMI* periodic limb movement index, *REM* rapid eye movement sleep, *RSWA* REM sleep without atonia

From other clinical characteristics, 7 patients (12.5%) were smokers, smoking between 10 and 40 cigarettes per day, 49 (87.5%) were never daily smoker. 36 (64.3%) were occasional drinkers, but they did not drink while being on the psychoactive medication except of 4 individuals. None of our patients confessed regular alcohol abuse. Patients did not report any recent or ongoing substance abuse. We found autoimmune diseases in 12 (21.4%) of our patients, 8 of them had Hashimoto’s thyroiditis, 2 psoriasis, 1 diabetes mellitus type I, 1 rheumatoid arthritis. In 8 (14.3%) of the patients we found positive perinatal history (6 of them reported asfyxia, two were born premature). Most of our patients achieved at least secondary school education level, 19 (33.9%) of them had BC degree or above, 33 (58.9%) finished secondary school and only 4 (7.1%) of our participants finished only a primary school. Considering the fact that our patients often planned to continue their education proces after finishing the treatment and due to a small sample size in one of our groups it was rather difficult to stratify them based on the highest level of education they achieved so far. The last factor of interest was family history of psychiatric disease. We found such disease in 18 cases (32.1%) in mother (1x dementia, 3x anxiety disorder, 12x depression, 2x alcohol abuse), 15 times (26.8%) in father (2x depression, 13x alcohol abuse), 12x (21.4%) in siblings (5x anxiety disorder, 5x depression, 2x alcohol). None of these clinical factors significantly influenced REM-sleep muscle activity.

### RSWA and medication

Table 2 shows patients’ medication in detail. 19 (33.9%) patients were currently on SSRI (selective serotonin reuptake inhibitors), 15 (26.8%) on SNRI (selective norepinephrine reuptake inhibitors), 9 (16.1%) on NaSSA (noradrenergic and specific serotonergic antidepressants), 14 (25%) on antipsychotics and 13 (23.2%) on mood stabilizers. 8 (14.28%) of the patients were prescribed anxiolytics, but they were taken only ad hoc and not 3 day before polysomnography. None of them was on Z-drugs. Four patients (7.1%) took no antidepressant treatment. Fifteen patients (26.8%) were on 1 category of medication, 23 (41.1%) patients were on 2 catagories of medication and 14 (25%) were on 3 classes. The more categories of antidepressants were taken, the higher the total amount of disturbed muscle atonia. However, this trend was not statistically significant (Spearman’s rho = 0.226, *p* = 0.093).

**Table 2:** Psychoactive medication

Medication class	Medication name	# cases	Doses (mg)
**Antidepressants**			
SSRI	Escitalopram	8	10–30
	Sertralin	5	100–300
	Citalopram	3	10–40
	Fluoxetine	2	60
	Paroxetine	1	40
		Total 19	
SNRI	Venlafaxine	15	150–300
SARI	Trazodone	16	50–300
NaSSA	Mirtazapine	6	15–45
	Mianserin	3	30–60
Other ADs	Vortioxetine	1	10
	Agomelatine	5	25–50
		Total 46	
**Antipsychotics**			
FGA	Chlorprotixen	1	15–30
SGA	Quetiapine	10	100–400
	Olanzapine	2	2,5–10
	Aripiprazol	1	15
		Total 14	
Anticonvulsants			
Mood stabilizers	Lamotrigine	5	100–300
	Valproate	3	1000–1500
Other AEs	Pregabaline	5	75–450
		Total 13	

Legend: *SSRI* selective serotonin re-uptake inhibitors, *SNRI* selective noradrenalin re-uptake inhibitors, *SARI* serotonin antagonists and reuptake inhibitors, *NaSSA* noradrenergic and specific serotoninergic antidepressants, *AD* antidepressants, *FGA* first generation antipsychotics, *SGA* second generation antipsychotics, *AE* anitepileptics

We analyzed potential correlations between the use of particular classes of medication and the degree of muscle atonia disruption. We did not find any significant correlation between RSWA% and the use of SSRI as a class as well as for particular drugs. However, we found a moderate positive correlation between SNRI use and degree of disturbed muscle atonia (Spearman’s rho = 0.332, *p* = 0.015).

Some relationship was also found between use of particular type of medication and RSWA %. There was a weak positive correlation between use of venlafaxine and RSWA% (Spearman’s rho = 0.292, *p* = 0.029) and quetiapin (Spearman’s rho = 0.289, *p* = 0.030). Other correlations were not significant or were not to be proved due to a small sample sizes.

### RSWA and psychiatric diagnoses

We devided the patients’ cohort into groups according to diagnoses. The precise list of diagnoses and their frequencies in our cohort can be found in Table 3. Thirty-five patients (62.5%) were diagnosed with mood disorder, 21 (37.5%) with anxiety disorder and 12 (21.4%) with personality disordes, that was typically the second diagnose. Four patients (7.1%) had a history of psychoactive substance abuse (2 alcohol, 1 benzodiazepine, 1 cocaine). We found no statistically significant correlation between specific diagnose and RSWA%.

**Table 3:** Psychiatric diagnoses

Psychiatric diagnoses	# cases
**PRIMARY DIAGNOSES**	
**Mood disorders**	
Recurrent depressive disorder	27
Bipolar disorder	6
Dysthymia	2
	Total 35
**Anxiety disorders**	
Obsessive-compulsive disorder	5
Panic disorder	5
Posttraumatic stress disorder	4
Generalized anxiety disorder	4
Phobic anxiety disorder	3
	Total 21
**COMORBID CONDITIONS**	
Personality disorders	12
Substance abuse	4
Eating disorders	2

### Current mental status

At BAI, 34 patients (60.7%) scored above 15 (moderate/severe anxiety) and at BDI 33 patients (58.9%) scored ≥19 (moderate/severe depression). We found a strong positive correlation between BAI and RSWA% (Spearman’s rho = 0.879, *p* < 0.001), and weak positive correlation between BDI and RSWA% (p = Spearman’s rho = 0.263, *p* = 0.05).

## Discussion

We found the degree of REM sleep atonia in younger (≤ 50 years) psychiatry patients to be very variable, ranging from very low to high degree of impairment. According the literature, there were several cut-offs established from different muscles included in the measurement [19–23]. We quantified the RSWA from mm. submentales and flexores digitorum superficiales, that would be most similar to SINBAR methods with estimated cut-off of 32% of REM sleep [19, 24]. If we applied this cut-off, we had 2 cleary defined RBD cases in our group of patients, but many other (35.7%) refered rare lifetime occurence of DEB that was not captured on v-PSG or occasional nightmares (62.8%). The occurence of DEB and RSWA were not always associated which could lead to false positive RBD diagnosis. Repeating the v-PSG to visualize both RSWA and DEB might be preferred for RBD diagnosis. We decided to score RSWA in 1-s-miniepochs instead of commonly used scoring criteria, because we believed that this strategy would be more exact and more appropriate in this specific cohort with highly supressed REM sleep.

### RSWA and age/gender, other clinical factors

The mean age of our participants was 38 years (age range between 26 and 50 years), with higher proportion of females. This could be due to more frequent psychiatric diagnoses in women, and also due to exclusion of 8 men suffering from obstructive sleep apnoea. This however, emphasasis the fact, that RSWA frequently occurs in young women, which is in line with currently available data, that describe relatively high occurrence of early onset RSWA/RBD in women [25], even suggesting a unique clinical profile of the condition. Patients who were diagnosed as early onset RBD were mainly female gender with significantly more past and ongoing psychiatric disorders and use of antidepressants [5]. Many of such patients are also middle-aged women suffering from autoimmune disease [11]. Nevertheless, other studies report different outcomes, finding no difference in RBD between male and female in middle-to-older general population [26] or even slight male predominance in patients under 50 years of age [27]. RBD is often thought to be under-recognised in women due to more subtle presentation [28]. The evidence is still inconclusive as it was also suggested that female subjects suffering from RBD present with comparable clinical features and prevalence of sleep-related injuries [29]. We did not find any association between increased RSWA% and age in our group of patients.

We found no significant difference in education level, but it was not possible to stratify this cohort much, because the majority of our patients had secondary school or higher education, with only 4 (7.1%) patients with completed primary education. The fact, that our patients did not report the use or abuse of alcohol could have been biased by participants‘possible tendency to confirm that they follow the recomendations regarding the use of psychoactive medication and alcohol consumption, which is not recomended in any case. Thus, we could argue that some patients intentionally lowered their daily alcohol consumption. We did not confirm this assumption by providing any objective alcohol levels testing. Due to patients‘long term stay in ward the possibility of their ongoing alcohol intake was very low.

Only 1 patient had past history of benzodiazepine abuse, 2 patients of alcohol and one of cocaine abuse. Literature shows that one of the RBD-subgroups can be middle-aged women with autoimmune disease [11], but we did not find any association between RSWA and history of autoimmune disease in our patients’ cohort, as well as in positive perinatal history or family history of mental diseases [30]. Because other studies confirm several risk factors for RBD in general population [26, 31], we hypothesized that it could reflect the epidemiological heterogeneity of RSWA/RBD.

### RSWA and medication

Concerning psychoactive medication, numerous drugs, including fluoxetine, venlafaxine, mirtazapine, paroxetine, clomipramine or sertraline, as well as antidepressants as a group, have been associated with exacerbation of RSWA/RBD [8, 12, 13, 32–34]. In their retrospective study, Winkelman and James described higher frequency of RSWA (RBD was not specifically assessed) in subjects taking serotonergic antidepressants compared to controls [8]. After withdrawal of SSRI the authors did not observe a complete resolution of clinical RBD symptoms and PSG abnormalitis [6, 32]. On the other hand, some RBD cases have been successfully treated with certain antidepressants [35] and withdrawal from certain antidepressants such as imipramine induced acute RBD [36]. Another study found no difference in atonia index between RBD patients taking or not antidepressants [37]. Moreover, some recent studies point out that also antipsychotics may play a role [26, 38] suggesting that dopaminergic modulation can influence RBD/RSWA.

Our patients typically took several antidepressants from different classes, that made it difficult to determine specific associations. Moreover, many of our patients were hospitalized because the only one medication (typically from SSRI class) did not have adequate effect on the severity of their psychiatric symptoms, and the cross-titration with SNRI was provided, that means that they were on relatively low doses. Nevertheless, we saw some trend in higher RSWA in patients who were on several psychoactive categories of medication and specificaly on SNRI and antipsychotics. According to the literature we expected higher RSWA also after current SSRI use, but likely, serotoninergic system alone is not enough to cause dysregulation of REM sleep atonia, and more profound and complex dysfunction of monoaminergic systems might be needed in order to cause clinically visible symptoms. Some evidence shows that the sole effect of SSRI agents is not convincing. A clinical epidemiological study conducted in a psychiatric out-patient setting found that the risk of developing RBD among those taking SSRI antidepressants was only 1 out of 20 [4] and another study suggests that serotoninergic system is not directly involved in the pathogenesis of RBD, even though an increased serotoninegic tone could unveil acute RBD in predisposed subjects [39, 40]. The noradrenergic system might also be involved in RBD pathogenesis [41] since noradrenergic antagonist drugs have been reported to induce RBD [42]. Our results suggest a complex interplay of different neurotransmitter systems including serotonin, noradrenalin and dopamin intervening in REM-on/REM-off balance. However, methodological issues could play a role, as several studies that confirmed the effect of SSRI were based on measurements of RSWA from mm. tibiales muscles, in contrast to our study [8, 12].

### RSWA and psychiatric diagnosis

Finaly, there is an ongoing controversy regarding the relationship between psychiatric illness itself as an etiological factor in RBD. Several studies showed potential relationship between RBD and severe stress or post-traumatic stress disorder (PTSD) [43–45]. It has been hypothesized that repeated traumas increase noradrenaline turnover, leading to its depletion in the LC, which in turn exerts an inhibitory action on the cholinergic laterodorsal tegmental LDT nucleus, tj. LC dysfunction might underlie both these disorders [43]. The evidence is however limited. Other studies did not find any clinically significant sleep disorder or typical pattern of sleep disturbance detectable by standard polysomnography in PTSD patients [46].

Wing et al. suggests that depression is a significant risk factor in predicting PD in iRBD patients [47]. On the other hand, McCarter claims that antidepressants but not depression appears to have a mediating role in promoting RSWA, given that only patients taking antidepressants had significantly higher muscle activity than psychiatric patients who are not treated with antidepressants [13]. Our results showed that more than given diagnoses, the actual mental state was important for patients’ RSWA%.

### RSWA and mental state

We found a positive correlation between BAI/BDI and REM sleep without atonia in our cohort of psychiatric patients. Lam et al. even described that psychiatric RBD patients had higher scores over BDI and HADS anxiety subscale (near statistical significance), HADS anxiety subscale showed significant difference between psychiatric and typical RBD cases [48]. Another study came with similar findings, declaring significantly more prominent symptoms of depression and anxiety in psychiatry RBD patients with higher Hospital Anxiety and Depression Scale and Beck Depression Inventory [49]. They claim, that there could be other individual susceptibility and clinical factors that increase the risk of developing RBD symptoms upon the onset of psychiatric illnessess and usage of antidepressants.

There are some limitations that could be mentioned. First is small number of cases on individual antidepressant agents. Unfortunatelly obtaining bigger sample size was not possible in our hospitalized psychiatric patients as they typically needed potent or multiple treatment. None of our patients presented motor or cognitive abnormalities during neurological examination. However, other biomarkers such as autonomic and specific sensory testings were not provided. Unfortunately, our results should also be interpreted with regard to the fact that we are not able to provide data obtained from control group.

In terms of RSWA assessment, 1-s miniepoch RSWA scoring may be a limitation for comparisons between studies. Nevertheless, we believe that in this specific sample with highly supressed REM sleep scoring in 3-s miniepochs could falsely increase the percentage of disturbed muscle atonia. Our patients underwent only one night in sleep laboratory, thus we could not underpin eventuall night-to-night variability in REM sleep atonia. Moreover, as we did not analyze the activity of anterior tibialis muscle, we are not able to confront our findings with current normative data for adult [50]. This would still be difficult as the authors of the study excluded patients who were receiving antidepressant medication during the time of the study. To our knowledge, normative data for psychiatric cohort are still missing.

## Conclusions

Isolated REM sleep without atonia in younger psychiatry patients is associated with psychoactive medication as well as current mental status, more that given diagnoses, but these factors are in most cases not sufficient to cause RBD. Further follow-up studies are needed to clarify whether current mental state, antidepressant use or other underlying factors might precipitate full-blown RBD. It is also not possible to apply the above-mentioned findings to older aged patients with RBD. Our findings indicate the heterogeneity of RBD spectrum disease. It also has many important public health implications since antidepressants are among the most commonly prescribed medications, and physicians should be aware of the potential for RSWA and subsequent injurious dream-enactment behaviours associated with these medications.

## Data Availability

The datasets used during the current study are available from the corresponding author on reasonable request.

## Abbreviations

AASM: American Academy of Sleep Medicine
AHI: Apnoea-hypopnoea index
BAI: Beck anxiety inventory
BDI-II: Beck depression inventory, second version
DEB: Dream-enacting behaviour
ECG: Electrocardiography
EEG: Electroencephalography
EMG: Electromyography
EOG: Electrooculography
FDS: Flexor digitorum superficialis muscle
LC: Locus coeruleus
LDT: Laterodorsal tegmentum nucleus
OSA: Obstructive sleep apnoea
PLMI: Periodic limb movements index
PLMS: Periodic limb movements in sleep
PTSD: Post-traumatic stress disorder
RBD: REM sleep behaviour disorder
REM: Rapid eye movement sleep
REMP: REM period
RSWA: REM sleep without atonia
SSRI/SNRI: Selective serotonin/noradrenalin re-uptake inhibitors
TA: Tibialis anterior muscle
v-PSG: Video-polysomnography
